# Reducing Anemia Prevalence in Afghanistan: Socioeconomic Correlates and the Particular Role of Agricultural Assets

**DOI:** 10.1371/journal.pone.0156878

**Published:** 2016-06-06

**Authors:** Artemisa Flores-Martinez, Giacomo Zanello, Bhavani Shankar, Nigel Poole

**Affiliations:** 1 SOAS, University of London and Leverhulme Centre for Integrative Research in Agriculture and Health, London, United Kingdom; 2 School of Agriculture, Policy and Development, University of Reading, Reading, United Kingdom; The Pennsylvania State University Hershey Medical Center, UNITED STATES

## Abstract

This research aims to examine the socio-economic correlates of anemia in women, and potential sources of iron in household diets in Afghanistan. It also examines whether ownership of agricultural (particularly livestock) assets and their use in food production has a role in alleviating anaemia, especially where local markets may be inadequate. We analyse data from the 2010/11 Afghanistan Multiple Indicator Cluster Survey, estimating a logistic regression to examine how anemia status of women is associated with socio-economic covariates. A key result found is that sheep ownership has a protective effect in reducing anemia (prevalence odds ratio of sheep ownership on anemia of 0.83, 95% confidence interval (CI): 0.73–0.94) after controlling for wealth and other covariates. This association is found to be robust to alternative model specifications. Given the central role of red meat in heme iron provision and absorption of non-heme iron, we hypothesise that sheep ownership promotes mutton consumption from own-production in a setting where market-sourced provision of nutritious food is a challenge. We then use the 2011/12 National Risk and Vulnerability Assessment household data to understand the Afghan diet from the perspective of dietary iron provision, and to understand interactions between own-production, market sourcing and mutton consumption. Sheep ownership is found to increase the likelihood that a household consumed mutton (odds ratio of 1.27, 95% CI: 1.15–1.42), the number of days in the week that mutton was consumed (prevalence rate ratio of 1.24. 95% CI: 1.12–1.37) and the quantity of mutton consumed (7 grams/person/week). In the subsample of mutton consumers, households sourcing mutton mostly from own production consumed mutton 1.5 days more frequently on average than households relying on market purchase, resulting in 100 grams per person per week higher mutton intake. Thus this analysis lends support to the notion that the linkage between sheep ownership and anemia risk is at least partly due to consumption arising from own-production in the presence of market incompleteness.

## Introduction

Anemia is a nutrition problem of global importance. Based on data from 107 countries worldwide, Stevens *et al*. [1] estimated that 29% of non-pregnant women (translating to 496 million non-pregnant women), 38% of pregnant women (translating to 32 million pregnant women) and 43% of children (translating to 273 million children) suffer from anemia. Analysis of data from the Global Burden of Disease project shows that 68 million years lived with disability (YLD) were attributable to anemia in 2010 [2]. Women and children are particularly susceptible to anemia, which has been linked to increased maternal mortality and morbidity, low birthweight, impaired cognitive development and lowered labour productivity, especially in the developing world [3–5]. Iron deficiency is the most common cause of anemia, although other nutritional deficiencies and various disorders affecting erythrocyte production and function are also contributory factors [6].

Iron-deficiency anemia control, and micronutrient malnutrition programming more generally, has historically relied predominantly on supplementation and fortification, whilst ‘food-based’ approaches have received correspondingly less attention [7]. However, the last few years have witnessed a surge of interest in understanding agriculture-nutrition linkages and shaping agriculture and food sector initiatives to achieve nutrition outcomes [8]. Significant investments have been made in developing biofortified varieties of key crops, including orange-fleshed sweet potato (OFSP), iron-rich beans and pearl millet.

However, while patterns of household agriculture are widely expected to impact nutrition and health of vulnerable groups, the evidence base for a positive impact of household food strategies on nutritional and health status, albeit growing, is still limited. A review by Berti *et al*. [9] considered the impact on nutritional status, including diets, anthropometry, biochemical indicators and morbidity, of a range of 30 agricultural interventions. They found that while most interventions increased food production, they did not necessarily improve nutritional outcomes. Leroy and Frongillo [10] reviewed the impact of livestock interventions on nutritional status as well as on intermediate outcomes such as production and dietary intake, finding positive impacts on production. The evidence for impact on nutritional status was limited, and the impact pathways—direct or indirect via sales and income effects—were unclear. Similarly, Webb-Girard *et al*.'s [11] review of 27 interventions found significantly improved dietary patterns but they were unable to identify strong associations with improved anthropometry, vitamin-A status, anemia and morbidity. A review by Masset *et al*. [12] found that agricultural interventions had positive impacts on consumption of nutritious foods but evidence for improvements in child nutrition status was unclear. Several of these reviews highlighted methodological shortcomings in the reviewed studies. The studies by Leroy and Frongillo and Webb-Girard, *et al*. highlighted the particular potential of livestock interventions and animal source foods to improve nutritional outcomes, but the evidence base remains limited.

South Asia is the region bearing the highest burden of anemia in general, and iron-deficiency anemia in particular. In 2010, 37.5% of the global anemia YLD originated in South Asia, with about 55% of anemia in the region attributable to iron deficiency [2]. In India, the anemia prevalence rate is about 50% for non-pregnant women, and as high as 85% for pregnant women [13]. Pakistan’s 2011 National Nutrition Survey showed that about 51% of both pregnant and non-pregnant women were anemic [14]. Conflict and instability has meant nutrition in Afghanistan has been much less researched than the rest of South Asia. A World Bank publication [15] summarises knowledge about the nutrition situation in Afghanistan. Using data from the National Nutrition Survey (NNS) of 2004, it reports an under-5 stunting rate of 60.5%, and anemia prevalence amongst non-pregnant women of 25% [15]. Summary results from a National Nutrition Survey conducted in 2013 have been reported recently in Varkey *et al*. [16] and Ministry of Public Health & UNICEF [17], and show that under-5 stunting prevalence has dropped to 40.9%. Anemia prevalence amongst women of reproductive age, including both pregnant and non-pregnant women, was 40% (the dataset is not publicly available, however). A study of 60 households in Northern Afghanistan by Levitt *et al*. [18] reports anemia prevalence amongst women (including pregnant and non-pregnant) of 25% in study households.

First, this paper aims to improve the scarce evidence base on the socio-economic drivers of anemia in women, and sources of iron in household diets in Afghanistan. Second, we ask whether ownership of agricultural assets, particularly livestock, and their use in food production, have a role in alleviating anemia, especially in areas where there local markets are scarce. In Afghanistan, where conflict and difficult terrain constrain market connectivity [19], this becomes a compelling association to examine. The connection between livestock ownership and nutrition outcomes, explored recently in research set in Ethiopia [20] and Uganda [21], has potentially important nutrition policy and programming implications. By answering this question, we also contribute to a larger literature examining how socioeconomic aspects of agricultural livelihoods have implications for nutrition in developing agrarian economies.

First, we analyse data from the Afghanistan Multiple Indicator Cluster Survey (AMICS) for Afghanistan conducted in 2010/11, estimating a logistic model to examine how anemia status of adult women is associated with a range of socio-economic and demographic covariates. Special attention is paid to the relationship between anemia and agricultural asset ownership. A number of model robustness checks is carried out. Since the MICS dataset does not contain information on food consumption, we then turn to analysis of National Risk and Vulnerability (NRVA) data from 2011–12. The NRVA contains detailed information on household food consumption in Afghanistan. We use it to examine Afghan diets from the point of view of dietary iron provision and the ways in which these foods are sourced (own-production, market purchase, *etc*.). The NRVA data allow us to follow-up key links between agricultural asset ownership and anemia outcomes uncovered by the AMICS data with a complementary analysis of how asset ownership and the sourcing of food might influence food consumption of particular relevance to dietary iron intake.

## Setting, Data and Methods

### Setting

Afghanistan, where this research is set, ranked 171 out of 188 countries in the UN Human Development Index, the lowest of any country outside Sub-Saharan Africa [22]. In 2011–12, 36 percent of the population in Afghanistan was under the poverty line [23]. Most economic and growth indicators show very slow recent progress [24]. Agriculture is the mainstay of the predominantly rural Afghan society: 80% of the total population and 90% of the poor live in rural areas and agriculture accounts for 40% of the labour market and about one-quarter of GDP [25].

Only about 12% of Afghanistan’s land area is arable, and the agriculture sector is overwhelmingly comprised of small producers engaged in traditional cultivation, often with a substantial subsistence element. Table 1 below provides some basic information on farming enterprises.

**Table 1:** Agriculture in Afghanistan.

Households owning irrigated land (%)	38
Households owning rain-fed land (%)	17
Households owning a garden plot (%)	13
Households owning cattle (%)	39
Households owning goats (%)	29
Households owning sheep (%)	31
Households owning chicken (%)	44
Median size of owned irrigated land (hectares)	0.6
Median size of owned rain-fed land (hectares)	1.4
Median size of owned garden plot (hectares)	0.2

Source: adapted from Central Statistics Organization (2014). *National Risk and Vulnerability Assessment 2011–2012*. *Afghanistan Living Conditions Survey*. Kabul, CSO, p.ii.

Wheat is the dominant farm enterprise. Afghanistan is amongst the world’s highest per capita wheat consumers, with 57% of average household calories deriving from wheat [25]. Rice, maize and barley are the other commonly grown crops. Livestock are integral to the household economy, and the vast majority of Afghan households hold some livestock (cattle, sheep, goats or chicken).

### Afghanistan Multiple Indicator Cluster Survey 2010–11 data

Our analysis of the drivers of anemia uses data from the AMICS 2010–11. AMICS 2010–11 data are nationally representative, with the overall sample drawn on the basis of a stratified two-stage sampling procedure and covering 22,053 women from 13,314 households visited across Afghanistan’s eight regions [26]. A subsample of 50% of the households in the sample (all households from odd-numbered clusters) was chosen for administration of the hemoglobin test amongst women aged 15–49. The dataset contains haemoglobin information for 9199 adult women. We considered any haemoglobin readings higher than 24 g/Dl as outliers, and dropped those data points, which left a sample of 9174 women for this analysis. As a sensitivity check, we also considered a lower threshold for outliers at 16 g/Dl, repeating our analysis for this more restricted sample of 8712 women. AMICS also collected a range of socio-demographic and health-related information, with separate questionnaires for women, children and the household unit. However, there is no food intake information available in the AMICS dataset.

### National Risk and Vulnerability Assessment 2011–12 data

NRVA 2011–12 is a nationally representative survey of the living standards of 20,828 households that was undertaken by the Central Statistics Organization of Afghanistan [27]. Household selection for NRVA 2011–12 was based on a stratified sampling procedure with a two-stage cluster design per stratum [27]. In addition to a 15 section household questionnaire covering aspects such as agricultural production and household assets, separate male and female modules probed specific areas where either the household head or their spouse was the main decision-maker. Included in the female module is seven-day recall information for the household on the quantity and sourcing of more than ninety foods typical to the Afghan diet.

Intra-household distribution of household food consumption is not captured by the NRVA, however. From household food consumption information we estimated per capita consumption based on the number of meals household members had at home, controlling for the number of guests. Although such an approach provides estimates of per-capita consumption, the data do not allow adjustment for intra-household allocation of food. After deletion of observations with missing values, the sample used in the analysis included 20,193 households.

Both the AMICS and the NRVA are nationally representative, with the AMICS designed to also be representative of each of Afghanistan regions, and the NRVA designed to also be representative at the province level. But they cover different time windows (AMICS was conducted in 2010–11 while NRVA was conducted in 2011–12), and did not sample the same households. Thus there is no scope to directly link the two datasets, and our analysis was conducted separately on the two datasets, with insights brought together in discussion.

### Logistic regression

We estimated logistic regressions to explain anemia status amongst women aged 15–49. After deleting outliers and observations with missing values from the subsample of the AMICS dataset with hemoglobin concentration (Hb) values, we retained information on 9174 women for analysis, comprising 904 pregnant and 8270 non-pregnant women. We pooled the pregnant and non-pregnant women samples in the modelling and included an indicator for pregnancy status as a covariate. In accordance with WHO guidance [28], anemia was defined on the basis of Hb lower than 12 grams/decilitre in non-pregnant women, and Hb less than 11 g/dl in pregnant women. We also conducted a sensitivity analysis comparing results from the pooled sample with results from the sample restricted to non-pregnant women.

An additional consideration of potential importance to Hb in this setting is altitude. A large proportion of Afghanistan’s population lives at elevations in excess of 1000 metres above sea level. Oxygen saturation of blood declines with altitude [29], and adjustments to Hb or the cut-offs for determining anemia prevalence are required to account for this. Sullivan *et al*. [30] present precise adjustment information that could equivalently be applied either to Hb values or to Hb cutoffs for anemia. However, the AMICS dataset does not record altitude information or geographical coordinates that would allow for altitudes to be inferred at individual level. The most disaggregate geographical information provided is on province. Hence, we adopted the following strategy: we compiled altitude information for the capital of each province and, for the logistic regression, adjusted the Hb of each individual in the sample based on the altitude of their provincial capital and the adjustment factors provided in Sullivan *et al*. [30]. Since altitudes can vary within a province, and our adjustment is based on altitudes of provincial capitals, which are typically located in valleys or relatively low-lying areas, our adjustment estimates may be regarded as lower bounds of fully adjusted Hb values. For the summary statistics and logistic regression components, we simultaneously present results based on both unadjusted as well as adjusted data.

The covariate set was informed by previous studies modelling anemia status amongst adult women, particularly those set in South Asia [31–33]. Categories of covariates relevant to explaining the modelled dependent variables were identified from this literature, and variables available in the AMICS dataset that matched these categories were shortlisted. Mid Upper Arm Circumference (MUAC) measurements were available in the survey, and was considered as a covariate. However, MUAC information was not collected from pregnant women and was additionally missing for about 10% of non-pregnant women, and hence this variable was dropped from the analysis. An index of vehicular assets (not included in the separate wealth/asset index provided with the dataset) was initially included in the analysis as an additional indicator of wealth, but was subsequently discarded due to difficulties arising in interpretation. Dropping this variable resulted in little change to the estimates. Collinearity diagnostics were performed on the final choice of covariates, and identified no significant multicollinearity issues. The shortlisted variables were all entered into the final regression model, without undertaking stepwise procedures for inclusion or deletion of variables.

Socio-economic covariates used in the chosen specification include measures of education and wealth. The primary wealth measure used is based on a wealth index (asset index) score in the dataset, which was created using a principal components procedure based on the type of water source in the household, the sanitation facility, house characteristics including roof, floor and wall types, the ratio of people to sleeping rooms, cooking fuel type, and ownership of durable appliances including refrigerator, TV and radio [26]. We classified households on the basis of the wealth quintile they fell into, using wealth quintile membership in our regression to aid interpretation. The ownership of agricultural land and cattle, horses, goats, sheep and poultry was captured in a set of dummy variables. Demographic variables include age, family size and number of children under five. In addition, we included variables indicating whether the woman has already had three or more children, and whether she has given birth within the last two years, to account for nutritional depletion arising from frequent births and recent birth. Unfortunately, the dataset did not contain information that would enable control for early or closely spaced pregnancies [34]. Control for environments encouraging infection were introduced in the form of variables representing drinking water treatment and toilet access. Cultural and geographical variations were captured in variables representing rural/urban location, region and ethno-linguistic group. Model fit was assessed using the Hosmer-Lemeshow chi-squared test [35] with the number of groups used in the test determined on the basis of the recommendations of Paul *et al*. [36].

To test the robustness of the logistic regression results, and in particular the key associations between agricultural assets and anemia, we checked the results against those from a different empirical framework, unconditional quantile regression. Quantile regression models the continuous variable underlying anemia status, *i*.*e*. Hb, directly, but allows for the association of covariates with Hb to vary across the distribution of Hb. The same covariate set used in modelling the logistic regression on anemia status was used in the quantile regression on Hb, enabling a comparison to gauge robustness. A series of other robustness checks was also carried out, including restriction to a sample of only non-pregnant women, Bonferroni-adjusted p-values for multiple testing, and comparison of a series of models with varying extents of parametrization using Akaike Information Criterion (AIC).

### Diets and sources of iron in Afghanistan

The NRVA data were first analysed to provide a basic characterisation of diets in Afghanistan and obtain an understanding of the potential dietary sources of iron. The NRVA household consumption data on various foods were converted into grams per capita per day measures. The foods were then aggregated into categories to provide a broad picture of diets.

### Regressions on factors determining food sourcing

Following up a key finding from the logistic regression analysis that anemia status is negatively related to household sheep ownership, we used the NRVA data to ask what relationship sheep ownership has with sheep meat (mutton) intake. We estimated a Logit model of whether mutton is consumed or not, a Poisson count model of the number of days of mutton consumption per week, and an OLS regression estimating household per capita mutton weekly consumption quantity. With each of these regressions, we also additionally explored the interaction between sheep ownership and the absence of markets. Since intra-community bartering may be another means of provisioning food [20], we also explored whether it is actually community (Shura) level ownership of sheep that matters rather than individual ownership or market presence.

For each food consumed by a household, the NRVA also provides information on the source of the food -own production (household consuming its own agricultural production), market purchase, *etc*. We queried the NRVA database regarding the sourcing of different food categories and estimated regressions to examine the interplay between own-production and market sourcing of food in enabling household mutton intake, for the subsample of households who reported positive mutton consumption.

In specifying the above regression models of mutton intake and sourcing, we drew on past literature specific to food security and intake in Afghanistan [19, 37], as well as literature linking agricultural involvement and dietary outcomes [20, 21, 38]. Categories of covariates relevant to explaining the modelled dependent variables were identified from this literature, and variables available in the NRVA dataset that matched these categories were shortlisted. The previous work by D’Souza and Jollife [19, 37], based on previous waves of the NRVA dataset, was particularly helpful in identifying relevant NRVA variables. Collinearity diagnostics were performed, and identified no significant multicollinearity issues. The shortlisted variables were all entered into the final regression model, without implementation of any stepwise procedures for inclusion or deletion of variables.

## Results

### Anemia prevalence

Table 2 shows anemia prevalence in the sample, based on both the unadjusted data as well as data with Hb adjusted for altitude. Based on unadjusted data, anemia prevalence is 20%, with a little more than half of anemic adult women being moderately or severely anemic. Overall anemia prevalence is similar in the pregnant (19%) and non-pregnant (20.5%) sub-samples. Adjusting the data for altitude in the provincial capital results in a substantially higher prevalence estimate of approximately 30%.

**Table 2:** Anemia prevalence in the AMICS sample of adult women in Afghanistan (n = 9174).

Category	Definition	Unadjusted*	Adjusted*
Anemic	Non-pregnant: hb < 12 g/dl; Pregnant: hb < 11 g/dl	20.3%	29.4%
Mildly anemic	Non-pregnant: 11≤hb<12 g/dl; Pregnant: 10≤hb<11 g/dl	10%	13.6%
Moderately anemic	Non-pregnant: 8≤hb<11 g/dl; Pregnant: 7≤hb<10 g/dl	9%	12.9%
Severely anemic	Non-pregnant: hb<8 g/dl; Pregnant: hb<7 g/dl	1.4%	2.8%

* ‘unadjusted' does not account for elevation. 'Adjusted' adjusts Hb for altitude of provincial capital based on Sullivan *et al* (1998).

### Summary statistics

Table 3 reports summary statistics for the covariates included in the regression analysis, for the sample as a whole and for the anemic and non-anemic groups of adult women. Both altitude-adjusted and unadjusted data are shown. Altitude adjustment makes a difference to how anemia prevalence relates to some covariates. In particular, the spatial distribution of anemia prevalence is altered somewhat with adjustment for altitude, with higher prevalence computed for the mountainous Central and Central Highland regions, and lower prevalence for the lower elevation areas in the Southwest and Northwest. As noted before, our adjustment for anemia based on altitudes of provincial capitals, though an improvement on unadjusted data, is likely to underestimate anemia in more mountainous regions. Thus anemia prevalence in the Central Highlands, Central Regions and Northeastern regions in particular is likely to be higher than estimated here.

**Table 3:** Summary statistics of covariates in the AMICS sample of adult women (n = 9174), by anemia status.

		Altitude unadjusted		Altitude adjusted	
Variable	Mean (s.d.) or %	Non-anemic^a^	Anemic^a^	Non-anemic^b^	Anemic^b^
Age in years	27.0 (9.5)	26.8***	27.9***	26.8***	27.5***
*Education*:					
No schooling	77.3%	76.3%***	81.2%***	76.0%***	80.4%***
Primary schooling	8.3%	8.4%	8.0%	8.6%	7.8%
Secondary + schooling	14.4%	15.3%***	10.7%***	15.4%***	11.8%***
*Household head’s education*:					
Head: no schooling	62.0%	61.1%***	65.5%***	61.2%**	64.1%**
Head: primary schooling	12.3%	12.3%	12.3%	12.5%	11.9%
Head: secondary + schooling	25.6%	26.5%***	22.2%***	26.3%*	24.1%*
Currently Pregnant	9.9%	10.0%	9.3%	10.0%	9.5%
Gave birth in last two years	24.2%	23.1%***	28.4%***	22.8%***	27.6%***
Has 3+ children	47.4%	46.2%***	52.0%***	46.3%**	50.0%**
Number of household members	9.2 (3.9)	9.2	9.3	9.1***	9.5***
Number of under-5s in household	1.4 (1.2)	1.4	1.4	1.3***	1.5***
*Language/ethnicity*					
Dari speaker	49.6%	51.5%***	42.0%***	51.8%***	44.2%***
Pashto speaker	37.4%	37.0%	39.1%	37.3%	37.7%
Uzbek speaker	8.0%	6.4%***	14.2%***	6.5%***	11.7%***
Turkmen speaker	2.1%	1.8%***	3.4%***	1.5%***	3.7%***
*Wealth quintiles*					
Wealth quintile 1 (lowest)	20%	19%***	24%***	18%***	25%***
Wealth quintile 2	20%	20%	20%	20%	20%
Wealth quintile 3	20%	20%	20%	20%	20%
Wealth quintile 4	20%	20%	20%	20%	20%
Wealth quintile 5	20%	21%***	16%***	22%***	15%***
Drinking water is treated	19.5%	19.6%	19.4%	19.2%	20.3%
Home has electricity	52.8%	54.3%***	46.8%***	55.3%***	46.7%***
Household owns agricultural land	57.9%	58.1%	57.2%	57.8%	58.2%
Household owns cattle	48.4%	47.8%*	50.8%*	47.5%**	50.6%**
Household owns horses/donkeys	30.5%	29.8%**	33.2%**	29.8%*	32.2%*
Household owns goats	29.5%	29.5%	29.3%	29.9%	28.5%
Household owns sheep	30.3%	31.0%**	27.8%**	30.7%	29.4%
Household owns chicken	51.7%	51.2%*	53.8%*	51.2%	52.8%
Located in rural area	73.1%	72.0%***	77.1%***	71.4%***	77.1%***
*Region*:					
Located in Central Region	20.5%	23.4%***	9.3%***	23.4%***	13.6%***
Located in Central Highlands	7.3%	8.7%***	2.0%***	7.8%**	6.2%**
Located in Eastern Region	11.1%	10.9%	11.8%	11.9%***	9.1%***
Located in Northwest Region	13.3%	11.9%***	18.9***	12.1%***	16.3%***
Located in Northeastern Region	14.8%	11.7%***	27.1%***	11.9%***	21.9%***
Located in Southern Region	10.9%	11.7%***	7.8%***	11.2%	10.0%
Located in Southeastern Region	12.8%	12.4%**	14.5%**	11.5%***	16.0%***
Located in Western Region	9.3%	9.5%	8.7%	10.3%***	6.9%***

Covariate values by anemia status are simple bivariate descriptions. Tests of differences between anemic and non-anemic groups are t-tests for continuous variables, and large sample tests of differences in proportions for categorical variables.

*** denotes statistical significance at the 1% level,

** at 5% level and,

* at 10% level.

^a^Values presented for anemic and non-anemic categories are unadjusted for altitude.

^b^Values presented for anemic and non-anemic categories are adjusted for provincial capital altitude.

Both adjusted and unadjusted summary statistics display the following patterns. Anemic women are on average slightly older than non-anemic women, and are worse educated. Anemic women are more likely to have recently given birth and to already have given birth to 3 or more children. Anemia prevalence also displays strong ethno-linguistic and regional patterns. The anemic subgroup has a smaller proportion of Dari (Persian) speakers than the non-anemic subgroup. Correspondingly, the anemic subgroup contains a larger proportion of women living in the Northeast and Northwest, and a smaller proportion of women living in the relatively prosperous Central region of the country. The anemic are more likely to belong to the lowest wealth quintile and less likely to belong to the highest quintile than the non-anemic. The anemic group also contains a larger proportion of rural residents, but the distribution of agricultural assets by anemia status shows a mixed picture. On the one hand, sheep ownership is more likely amongst the non-anemic, at least on the basis of unadjusted data. On the other hand, cattle, horse and chicken ownership is more likely amongst the anemic as compared to the non-anemic. Land ownership is not statistically different across the two groups.

### Logistic regression

We turn to our regression analyses results to isolate associations between anemia and covariates of interest. Table 4 presents the results from the logistic regression applied to both altitude-unadjusted as well as adjusted data. The Hosmer-Lemeshow test’s null hypothesis of no difference between observed and fitted values of the outcome variable is not rejected at the 5% level for either model. Thus we find no evidence of a lack of model fit.

**Table 4:** Logistic regression results explaining anemia status in the AMICS sample of adult women in Afghanistan (n = 9174).

	Anemic (unadjusted)		Anemic (adjusted)	
	Odds Ratio	95% C.I.	Odds Ratio	95% C.I.
Age in years	1.040	(0.99–1.09)	1.043**	(1.00–1.09)
Age in years squared	1.000	(1.00–1.00)	0.999*	(1.00–1.00)
*Education (No schooling as reference)*				
Primary schooling	1.114	(0.90–1.37)	1.089	(0.91–1.31)
Secondary + schooling	1.007	(0.83–1.23)	1.040	(0.88–1.23)
*Household head's education (Head no education as reference)*				
Head primary	0.949	(0.80–1.12)	1.006	(0.87–1.17)
Head secondary plus	1.001	(0.87–1.16)	1.114*	(0.99–1.26)
Currently Pregnant	0.846*	(0.70–1.02)	0.884	(0.75–1.04)
Gave birth in last two years	1.256***	(1.09–1.45)	1.220***	(1.08–1.38)
Has 3+ children	1.071	(0.90–1.27)	1.008	(0.87–1.17)
Number of household members	1.017*	(1.00–1.04)	1.028***	(1.01–1.04)
Number of under-5s in household	0.955	(0.90–1.01)	0.984	(0.94–1.03)
*Language/ethnicity (Dari as reference)*				
Pashto speaker	1.357***	(1.15–1.60)	1.178**	(1.02–1.36)
Uzbek speaker	1.127	(0.92–1.37)	1.211**	(1.00–1.46)
Turkmen speaker	0.979	(0.70–1.36)	1.850***	(1.36–2.52)
*Wealth quintiles (quintile 1 as reference)*				
Wealth quintile 2	0.750***	(0.63–0.89)	0.594***	(0.51–0.69)
Wealth quintile 3	0.795**	(0.66–0.95)	0.615***	(0.52–0.72)
Wealth quintile 4	0.775**	(0.63–0.95)	0.563***	(0.47–0.68)
Wealth quintile 5	0.702**	(0.54–0.92)	0.520***	(0.41–0.66)
Drinking water is treated	1.123	(0.97–1.29)	1.113*	(0.99–1.26)
House has electricity	0.944	(0.82–1.09)	0.855**	(0.76–0.97)
Household owns agricultural land	0.903	(0.79–1.03)	0.897*	(0.80–1.01)
Household owns cattle	0.998	(0.86–1.16)	1.028	(0.90–1.18)
Household owns horses/donkeys	1.018	(0.88–1.18)	0.950	(0.84–1.08)
Household owns goats	0.922	(0.80–1.06)	0.839***	(0.74–0.95)
Household owns sheep	0.802***	(0.69–0.93)	0.830***	(0.73–0.94)
Household owns chicken	1.193***	(1.04–1.36)	1.070	(0.95–1.20)
Located in rural area	0.969	(0.80–1.18)	0.969	(0.82–1.15)
*Region (Central as reference)*				
Located in Central Highlands	0.603**	(0.41–0.89)	1.398***	(1.10–1.77)
Located in Eastern Region	2.278***	(1.75–2.97)	0.887	(0.70–1.12)
Located in Northwest Region	4.163***	(3.34–5.19)	2.358***	(1.96–2.83)
Located in Northeastern Region	5.939***	(4.75–7.43)	2.735***	(2.26–3.31)
Located in Southern Region	1.396**	(1.07–1.83)	1.292**	(1.05–1.58)
Located in Southeastern Region	2.678***	(2.12–3.38)	2.253***	(1.87–2.72)
Located in Western Region	2.503***	(1.95–3.21)	1.207*	(0.98–1.49)
Intercept	0.17***	(0.03–0.12)	0.060***	(0.09–0.32)
*Observations*	9174		9174	
*Hosmer-Lemeshow Chi-squared test statistic (675)*	651.29		*720*.*05*	
*Hosmer-Lemeshow p-value*	0.72		0.10	

Logistic regression odds ratios for anemia status based on altitude-adjusted and unadjusted haemoglobin values. We tested for multicollinearity in the models using Variance Inflation Factors (VIF). Variables in the models had a VIF of 1.83 (and none greater than 4.99). Covariates also include a dummy variable to capture a small number of observations that either had missing information for language/ethnicity or spoke a language other than Dari, Pashto, Uzbek or Turkmen. Robust standard errors.

*** denotes statistical significance at the 1% level,

** at 5% level and,

* at 10% level.

Directions to survey questions corresponding to variables are provided in S2 Supporting Information.

Taken together, results from the unadjusted and adjusted models indicate that neither the education of the woman nor that of her household head makes much difference to anemia status once other covariates are controlled for. This is in contrast to previous results from the region that show a negative relationship between educational attainment and anemia risk [31, 32]. Both models suggest that iron depletion from a recent birth event raises anemia risk. Once differential wealth and other covariates are controlled for in the regression, a major ethno-linguistic aspect is that Pashtun women are significantly more at risk of anemia than Dari women. Results from both models highlight the substantial regional heterogeneity in anemia prevalence even after control for wealth and other confounders. The anemia prevalence odds of women from the Northeast and North are respectively 5.9 and 4.1 times the prevalence odds of women in the Central region under the unadjusted model, and 2.7 and 2.3 times under the adjusted model. Women in the rest of the country are generally at higher risk of anemia than in Central Afghanistan.

Women from higher wealth quintiles are less likely to be anemic than women belonging to the lowest wealth quintile. Interestingly, once wealth and other covariates are controlled for, we find that none of the large agricultural assets (land, cattle, horses) has a statistically significant relationship with anemia status. In contrast, in both models sheep ownership shows a statistically significant relationship with anemia status even after control for a large number of wealth indicators. Sheep ownership is associated with a 20% reduction in prevalence odds for anemia under the unadjusted model, and a 17% reduction under the adjusted model. The relationships of goat and chicken ownership with anemia status are less consistent across the two models. Goat ownership is associated with a statistically significant reduction in anemia odds only under the adjusted model, while chicken ownership’s association with an increase in anemia odds is significant only under the unadjusted model.

#### Robustness checks

S1 Table presents results from applying the model to the non-pregnant sample only, which serves as a robustness check. The results do not change in any substantial way compared to the pooled sample results discussed above, indicating that the results are not skewed by pooling non-pregnant and pregnant samples.

A further check of robustness is provided by the unconditional quantile regression results reported in S1 Supporting Information. These reinforce many of the results from the logistic regression, but add a distribution-wide perspective. Education levels of the women and their household heads are not significantly associated with Hb. Recent birth, *i*.*e*. having given birth within the last two years, is found to have a particularly strong, negative association with Hb at the lower tail of the Hb distribution. Pashto speakers have lower Hb levels than Dari speakers at parts of the Hb distribution. The favourable Hb status of residents of the Central region compared to elsewhere in the country holds throughout the lower half of the Hb distribution.

Mirroring results from the logistic regression, the UQR results with respect to large agricultural assets (land, cattle and horses) also show little association with Hb along its distribution once wealth is controlled for. However, sheep and chicken ownership do display statistically significant relationships with Hb—the former positive and latter negative. But sheep ownership has a more consistent relationship than chicken ownership with Hb. Chicken ownership parameters are only significant at the 15^th^ and 25^th^ percentiles of Hb. On the other hand, sheep ownership shows a strong, statistically significant negative association with Hb throughout the lower half of the Hb distribution.

Multiple testing is a potential issue to be considered when hypotheses about a large number of variables are tested in a regression such as ours [39]. With a large number of hypotheses being tested, the probability of occurrence of one or more type I errors, where irrelevant variables appear significant, can become large. Making ‘Bonferroni’ adjustments to p-values or critical levels is one way to handle this. However, the need for Bonferroni adjustments is a hotly debated topic in many sciences. Some authors have refuted the need for adjustment, arguing that adjustment to reduce type I errors can only come at the cost of increasing the probability of type II errors where potentially important relationships are found to be statistically insignificant [40]. Others have argued that adjustment is needed only in selected circumstances, and that adjustment may not be appropriate in exploratory studies where potentially important effects may be uncovered for follow-up [41].

In this study, our primary mechanism to judge reliability is robustness of key results to alternative specifications, as detailed above. However, we also present Bonferroni adjusted p-values for the logistic model in S2 Table. The only variables that stay significant after adjustment are having a birth in the last two years, the second wealth quintile (compared to the first), being Pashto (compared to Dari), and regional dummies. None of the asset variables are significant, though it is worth noting that sheep ownership comes close to being significant at the 10% level.

In S3 Table, we provide further indication of: a) the acceptability of the model described in Table 4 compared to models containing fewer variables and b) the robustness of the key result on the negative association between sheep ownership and anemia. The table presents a series of nine alternative models, starting with the sparsest one only containing the sheep ownership covariate and adding sets of variables successively to reach the full model that was presented in Table 4. In each case, the odds ratio attached to the sheep ownership variable is presented, as well as the Akaike Information Criterion (AIC) statistic [42]. The AIC can be helpful in model selection, balancing goodness of fit with extent of parameterization. Results presented in S3 Table show sheep ownership to be statistically significant in all 9 alternative models with altitude-unadjusted data, and in 7 out of 9 models with adjusted data. The chosen model in Table 4 has the lowest AIC value among competing models, increasing confidence in the model.

### Diets and sources of iron in Afghanistan

Table 5 presents information on diets in Afghanistan. The dominance of wheat in Afghan diets becomes obvious from the table. At 142 kg/person/year Afghanistan has one of the highest per capita levels of wheat consumption in the world. At this level of consumption, a high percentage of iron intake is likely to derive from wheat flour. Vegetables and fruits that are important sources of iron in the Afghan diets include beans, peppers and apricots. However, the most heavily consumed fruits and vegetables in Afghanistan such as potato and melon are comparatively less dense in iron content. Animal source foods intake is dominated by dairy products, particularly fresh milk. Meat, an important source of iron, is consumed in modest amounts. Table 6 provides a comparative perspective on meat consumption, comparing Afghanistan to the region and selected neighbouring countries. Afghanistan is seen to have a much lower meat intake per capita compared to the Asian average and its relatively prosperous neighbour, Iran. Afghanistan’s meat intake compares favourably only to India where vegetarianism is a significant aspect. Notably, however, Afghanistan’s per capita sheep/goat meat consumption is higher than the Asian average and that of its closest comparator, Pakistan.

**Table 5:** Food Consumption in Afghanistan (kg/person/year) (n = 20,153).

Wheat flour	142.2
Animal source foods	67.1
Dairy	53.2
Meat (except fish)	11.9
Chicken	4.2
Bovine	4.1
Mutton	1.8
Goat	1.0
Other meat	0.8
Eggs	1.8
Fish	0.2
Vegetables	66.5
Other cereals	35.2
Fruits & nuts	33.8
Sugar & sweets	12.7
Oils & fats	12.3
Spices	11.1
Legumes	9.0
Beverages	5.6
Purchased nan	3.9

Calculated based on 7-day data from NRVA 2010–11 and raised to annual per capita estimates.

**Table 6:** Regional meat consumption per capita (kg/person/year).

Country / Region	Meat	Poultry	Bovine	Sheep/Goat
Afghanistan	11.9	4.2	4.9	2.8
Asia	31.9	9.7	4.5	1.9
Iran	38.5	23.6	8.1	6.6
India	5.1	2.4	1.7	0.6
Pakistan	14.9	2.0	8.1	2.5

Source: Afghanistan: Own calculations (shown in Table 5) based on data from the NRVA 2011–12; where bovine includes beef, veal, dried meat, liver and other meat. Asia and individual countries (average for 2011/12): Own calculation using consumption data from FAO (2012) and population data from UNDESA (2015). Pakistan poultry consumption estimate is drawn from the Household Income and Expenditure Survey of Pakistan. Regional data for total meat consumption includes pig meat.

Of course, while a knowledge of local diets provides a glimpse into key sources of iron intake, it is important to take into account that the bioavailability of heme and non-heme iron is substantially different. On average, about 40% of iron content in animal source foods is heme iron, and 60% is non-heme iron, whereas 100% of plant source foods is non-heme iron [43] The bioavailability of heme iron has been estimated to range between 15 and 35%, while the bioavailability of non-heme iron has been found to vary between 2 and 20% [44, 45]. Dairy, tea and phytate-containing foods such as grains and legumes are potentially important non-heme iron absorption inhibitors in this context [46, 18], whereas meat and ascorbic acid sources are key non-heme iron absorption enhancers [47]. The relatively high bioavailability of meat compared to non-heme sources of iron, and its ability, even in small doses, to enhance the bioavailability of non-heme iron [47] makes it a more important dietary factor in combating anemia than overall dietary contributions might suggest. On the other hand, the non-heme nature of their iron content and/or their roles as iron inhibitors [46, 19] may make certain foods such as wheat flour and legumes less effective in controlling anemia risk than their overall iron contributions might suggest.

The association we have found between sheep ownership and anemia status after controlling for a potential asset effect and the potential importance of meat intake in lowering anemia risk in this setting is consistent with own-production of consumed sheep meat having a role in lowering anemia prevalence. This is explored further in the regression results reported in Table 7 that model mutton consumption’s association with sheep ownership and other variables.

**Table 7:** Regression results: Sheep ownership and household mutton consumption in the NRVA sample of households in Afghanistan (n = 20,153).

	A. Mutton consumption (binary)			B. Num. days mutton consumption in a week			C. Per capita mutton consumption (Kg)		
	I	II	III	I	II	III	I	II	III
HH owns sheep	1.27***	1.005	1.183***	1.237***	0.946	1.148***	0.007***	-0.008	0.004*
	(1.15–1.42)	(0.76–1.33)	(1.06–1.32)	(1.12–1.37)	(0.75–1.19)	(1.03–1.27)	(0.00–0.01)	(-0.02–0.00)	(0.00–0.01)
No mkt in community	0.901	0.827**	0.887	0.885*	0.80***	0.870*	-0.007*	-0.012***	-0.008**
	(0.77–1.05)	(0.70–0.98)	(0.76–1.04)	(0.77–1.02)	(0.69–0.94)	(0.75–1.01)	(-0.01–0.00)	(-0.02–0.00)	(-0.02–0.00)
HH owns sheep * No mkt in community		1.322*			1.379**			0.016***	
		(0.99–1.77)			(1.08–1.76)			(0.00–0.03)	
Number of sheep in Shura (log)			1.077***			1.06***			0.002*
			(1.03–1.13)			(1.02–1.12)			(0.00–0.00)
*Hosmer-Lemeshow Chi-squared statistic (3251)*	3184.6	3219.15	3144.05						
*Hosmer-Lemeshow p-value*	0.78	0.64	0.90						

Model A reports odd ratios. Incidence-rate ratios are reported in Model B. Model C reports coefficients. Standard errors clustered at Shura level. Coefficient interval at 95% in parentheses.

*indicates significance at the 10% level,

**at the 5% level and,

***at the 1% level.

We tested for multicollinearity in the models using Variance Inflation Factors (VIF). Variables in the model had an average VIF of 2.01 (and none greater than 2.44). For each model we estimated three specifications, a basic model (I), a model with interaction term between ownership of sheep and the absence of market in the community (II), and with a variable proxying the number of sheep in the Shura (III). Each model also controls for a set of household characteristics (age and literacy of household head, household dependency ratio, numbers of adult males and females and children), rural residence, income (consumption) quintiles, wealth quintiles, survey timing (season of survey administration, and whether it was during Ramadan), and province dummies. Directions to survey questions corresponding to variables are provided in S2 Supporting Information.

Table 7 suggests a strong and consistent role for own-production of sheep in household mutton intake. Household sheep ownership is positive and significant at the 1% level in column 1 of each of the three models. Thus sheep ownership is associated with the decision to consume mutton, an increase in the number of days in which mutton is consumed in the week, and the per capita quantity of mutton consumption. There is also some evidence of the absence of markets reducing frequency and quantity of mutton consumption. In the interaction models (II), in each case the interaction term is positive and significant, with the non-interaction term for sheep ownership becoming very small in comparison to (I). This implies that household sheep ownership becomes important to mutton consumption decisions particularly in the absence of markets. Finally, in the models (III), the Shura-level sheep ownership parameter is positive and significant, but the household ownership stays positive and significant in each case, even if smaller than in comparison to (I). Thus community-level sheep ownership may enhance mutton consumption by providing bartering opportunities, but household sheep ownership remains important to consumption.

So far, our evidence linking sheep ownership to mutton intake (particularly in the absence of village markets) and anemia is suggestive of own-production of sheep being important. Is there any direct evidence that own-production of mutton/sheep meat is prevalent in Afghanistan? Table 8 presents self-reported sources of household consumption of animal source foods in the NRVA. Animal source food sourcing is heavily influenced by fresh milk, which is predominantly own-produced. The larger part of meat from small stock, *i*.*e*. sheep, goat and chicken, comes from purchases, but about a fifth of consumption is sourced from own production (direct consumption of household animal holdings). On the other hand, beef consumption, another importance source of iron from red meat, comes almost entirely from market purchase.

**Table 8:** Main sources of animal source foods in the NRVA sample of households in Afghanistan (n = 20,153) (%).

Main Source	ASF^a^	Beef	Mutton	Chicken	Goat	Fresh Milk
Purchase	54.4%	94.5%	80.6%	83.2%	69.0%	28.2%
Own production	39.9%	1.6%	13.0%	15.7%	20.7%	67.1%
Bartered / payment in kind	0.2%	0.1%	0.5%	0.1%	0.3%	0.2%
Borrowed / taken on credit	0.2%	0.3%	0.4%	0.4%	0.8%	0.1%
Received as gift	3.7%	1.2%	3.2%	0.3%	4.8%	3.6%
Food aid	1.0%	1.2%	1.0%	0.2%	4.0%	0.6%
Other	0.4%	1.1%	1.2%	0.1%	0.5%	0.2%

Population-weighted percentages based on frequencies.

^a^ASF stands for animal source foods.

Table 9 presents regression results modelling mutton intake on the sourcing of mutton, for the subsample of households that report positive mutton consumption. Own production (*i*.*e*. direct consumption of household animal holdings), in comparison to market purchase, is positive and strongly significant for both measures of consumption considered. The results indicate that all things, including wealth and income held equal, households sourcing mutton mostly from own production consumed mutton 1.5 days more frequently on average than households relying on market purchase, resulting in 100 grams per person per week higher mutton intake. The household sheep ownership variable is insignificant in the presence of own production (although the former is obviously a pre-requisite for the latter) and other sourcing variables. Taken together, these results suggest that own-production (direct consumption of animal holdings) arising from sheep ownership has a role in increased mutton intake. Thus the relationship between sheep ownership and increased mutton consumption may not arise solely from sheep ownership being an indicator of wealth or providing income generation. Converting the 100g/person/day higher intake into iron content without consideration of bioavailability or absorption factors [48] and comparing with the Recommended Daily Allowance (RDA) for iron among adult women of 18 mg/person/day [49], indicates that the increased intake translates into only a 2 to 3% contribution to RDA. However, the bioavailability of heme iron in mutton and its function in enhancing bioavailability of nonheme iron in the diet can imply a significantly larger effect on haemoglobin and anemia risk. This is consistent with a finding from neighbouring Pakistan that low red meat intake is associated with anemia among pregnant women [32].

**Table 9:** Regression results: Sourcing and consumption of mutton in the NRVA sub-sample of households reporting positive mutton consumption (n = 2721).

	A. Num. days mutton consumption in a week	B. Per capita mutton consumption (Kg)
HH owns sheep	0.969	-0.016
	(0.028)	(0.011)
*Source of meat (purchased as reference)*		
Own production	1.509***	0.102***
	(0.065)	(0.017)
Received as gift	0.975	-0.032**
	(0.072)	(0.016)
Food aid	0.971	0.078
	(0.110)	(0.080)
Other	1.105	0.029
	(0.124)	(0.032)
No mkt in community	0.947	-0.007
	(0.038)	(0.014)

Model A reports incidence-rate ratios. Coefficients are reported in Model B. Standard errors clustered at Shura level in parentheses.

*indicates significance at the 10% level,

**at the 5% level and,

***at the 1% level. We tested for multicollinearity in the models using Variance Inflation Factors (VIF). Variables in the models obtained an average VIF equals to 2.02 (and none greater than 4.89). Each model also controls for a set of household characteristics (age and literacy of household head, household dependency ratio, numbers of adult males and females and children), rural residence, income (consumption) quintiles, wealth quintiles, survey timing (season of survey administration, and whether it was during Ramadan), and province dummies.

## Discussion and Conclusion

The high prevalence of iron deficiency and anemia elsewhere in the South Asian region and Afghanistan’s large burden of women’s ill-health provides the rationale for this study’s focus on anemia amongst adult women. Approximately 20% of adult Afghan women in our sample (19% of pregnant women and 20.5% of non-pregnant women) were found to be anemic based on altitude-unadjusted data, 30% based on data adjusted for provincial altitude. This prevalence classifies the anemia problem amongst adult women as being of ‘moderate public health significance’ according to the WHO classification.

Anemia prevalence amongst adult women is thus lower in Afghanistan than in its neighbours to the east and north. However, the overall prevalence masks significant regional inequality, with high prevalence in the North East region bordering Pakistan and Tajikistan on the one extreme, and low prevalence in the Central Highlands and Central regions, including Kabul, on the other. This regional inequality is consistent with other development indices. Provinces in the North and Northeast regions, such as Badakshan, Balkh and Samangan are known to be have high incidences of poverty and food insecurity, especially in comparison to the relatively prosperous and well-endowed Central provinces such as Kabul, Kapis and Wardak [50]. Results reported here suggest that wealth and family planning outcomes, amongst other welfare indicators, are important correlates of anemia. The spatial distribution of these correlates may partly explain regional inequalities in anemia prevalence. However, our regression results show that some regional association with anemia prevalence persists even after controlling for wealth, family planning and other covariates. Our results further suggest that there are specific ethno-linguistic patterns to anemia risk. Of the principal ethno-linguistic groups, Pashtun women are disadvantaged compared to Dari speakers. Whether these regional and ethno-linguistic differences arise due to cultural and attitudinal differences, or dietary patterns or other factors, is worthy of future investigation. Such considerations, and the extent and nature of external donor intervention, may also play a role in explaining differences between anemia prevalence in Afghanistan compared to neighbouring countries, which is another interesting research question for the future.

The finding that pregnant women have lower odds of being anemic than non-pregnant women, although only statistically significant at the 10% level in the model unadjusted for altitude, is unexpected. Iron supplementation for pregnant women at antenatal care services may have a role to play in explaining this. Neither the AMICS nor the NRVA survey contains information on iron supplementation, and so this cannot be checked directly. However, Levitt *et al* [18] found that just over 50% of women in their sample had received iron-folate supplements during their last pregnancy. The World Bank review of nutrition in Afghanistan [15] reports that iron and folic acid supplementation is available through prenatal care services, and that care coverage is 36%. The influence of recent birth and household size on anemia prevalence underlines the importance of ongoing major efforts to mainstream family planning in Afghanistan [51]. While considerable progress has been achieved in the last decade, the use of modern family planning methods still stands only at 22%. Our results suggest that a lack of formal education is not by itself a significant barrier to combating anemia.

Turning to iron intake, whilst a variety of intervention options based on iron supplementation or fortification can be envisaged, achieving broad-based coverage for individual interventions can be a challenge in this setting. For example, fortifying wheat flour suggests itself as an attractive approach given the central importance of wheat flour in Afghan diets, and a fortification programme by the World Food Program, supported by the Micronutrient Initiative, has done just that. However, the private industrial flour mills covered under this initiative account for only 8% of urban flour consumption, and it is particularly challenging to fortify rural flour consumption in Afghanistan where most rural residents use small village mills [15]. In light of such challenges, there is a clear rationale for a prominent role for food-based approaches (dietary modifications) in the portfolio of strategies to address iron deficiency [7]. Neither the MICS nor the NRVA surveys included questions about flour fortification, and thus this information is missing in our estimates. However, given only 25% of the population is urban and flour fortification reached only 8% of urban households, we do not expect this missing information to bias our estimates in a significant way.

Many of the key foods in the Afghan diet, such as wheat flour, milk and legumes, are low in bioavailable iron contributions and/or are inhibitors of iron absorption. This elevates the importance of red meat in combating anemia given its contribution of heme iron and role as enhancer of iron absorption. Our results suggest that ownership of sheep is negatively associated with anemia status. This negative relationship between sheep ownership and anemia prevalence is robust to sensitivity checks and different specifications of the model. None of the other agricultural asset indicators show such a robust relationship with anemia across models.

Given the AMICS data are cross-sectional, we cannot be certain of a causal linkage between sheep ownership and anemia status. There is the possibility that the association may arise simply because sheep and other livestock are frequently stores of wealth in developing countries, and wealthier households are also likely to display better nutritional outcomes than poorer households. However, our regressions control for a large variety of assets and wealth indicators. This aspect, along with the preeminent role of meat consumption in providing heme iron and improving the bioavailability of non-heme iron even in small doses, is suggestive of own-consumption of sheep meat playing a role in improving Hb. This is consistent with results from elsewhere in the region emphasising the lack of meat in diets as a key cause of anemia in women [33].

The NRVA data have shown that, while market purchase is dominant as a source of animal source foods, consumption from own production is also important in Afghanistan. Patterns of sourcing shown in the NRVA data are consistent with what is known about the main uses of different livestock species in Afghanistan. The main use of cattle is for milk production, with beef own-production being minimal. The priority for chicken use is sale of eggs, and to a lesser extent, meat production. Sheep are mostly valued for their meat, with wool and milk provision being of secondary importance, while goats are valued most for their milk [52]. Sheep meat is usually preferred to goat meat [53], and is the most important source of meat calories [19]. Besides festivals, lamb slaughter often takes place in the autumn for production of dried mutton that can be preserved for longer.

Previous evidence reviews have explored the potential for livestock interventions to improve nutrition outcomes, but have found the evidence base to be very limited [10, 11]. In particular, clear evidence regarding the pathways (own production, increased income, caregiver time, *etc*.*)* through which improved animal production improves nutrition have been lacking. Our regressions based on NRVA data corroborate the notion that the linkage between sheep ownership and Hb concentration is at least partly due to consumption from own-production in the presence of market incompleteness. Sheep ownership is seen to be linked to greater mutton consumption after controlling for wealth and income, and to do so particularly when village markets are absent. More specifically, own production sourcing of mutton is associated with higher sheep meat consumption than market purchase.

However, it is important to note that this study’s finding that own-production of mutton may have a role to play in improving iron intakes and lowering anemia in Afghanistan should not be interpreted as downplaying the importance of market sourcing of nutrition. With 80% of mutton consumption as well as high proportions of beef, chicken and goat consumption arising from market purchase, market sourcing of animal food nutrition is very important in Afghanistan. Market development is a key pillar in long-term poverty alleviation [54] and nutritional improvement [38] strategies. Our results suggest that own-production has a complementary part to play in alleviating anemia, especially where market access is limited.

This study has several shortcomings. Each of the two datasets we use contains rich information on certain aspects of the Afghan population, but is deficient in other areas. Thus neither by itself allows full answering of the cross-sectoral questions of interest to this research. The AMICS dataset provides detailed information on human development indicators, including haemoglobin. However, it lacks food consumption data and only contains basic information on livelihoods and agriculture. The NRVA has detailed data on livelihoods and food consumption. However, it is a household-level dataset and does not provide an individual-level perspective, and does not contain information on nutrition outcomes. Direct linkage of the two datasets is not possible due to their different sampling procedures and time frames for data collection. Our analysis thus attempts to draw insights from separate analyses on each dataset that are then fused together in discussion. Our results are therefore only indicative, and we cannot claim robust evidence for the entire pathway from sheep ownership to mutton consumption to anemia status. The data challenge encountered here is typical of data disconnects that characterise research on agriculture-nutrition linkages, and underlines the need for integration of data collection efforts, bringing together living standards data collection (such as the NRVA) and health data collection (such as the MICS and DHS surveys) [8].

The binary representation of livestock ownership in our logistic regression is a significant limitation of this study. This variable attempts to proxy off-take, or more specifically, own-consumption. By not capturing livestock numbers, age and other parameters that may jointly proxy off-take or consumption, or indeed off-take or consumption directly, measurement error is introduced. These measurement errors are independent of anemia status, and are thus classifiable as a type of non-differential measurement bias [55, 56]. This is likely to bias the estimates towards the null value [55, 56] and thus the true relationship between sheep offtake/consumption and anemia is likely to be underestimated. This reinforces the call made above for integrated data collection where detailed agricultural and living standards data collection is combined with nutrition and health data collection to enable consistent and accurate assessments in the agriculture-nutrition space.

Furthermore, as a cross-sectional observational study, the ability to draw causal conclusions is inevitably limited. Thus, although the relationship we find between sheep ownership and mutton consumption and anemia controls for a range of potential confounders and appears robust to alternative model specifications, a claim about clear causal identification cannot be made. However, our results are at least suggestive of a need for further research to explore the potential contribution of policies and interventions targeted at broad-based improvement in red meat, specifically sheep meat, consumption in Afghanistan as a component of a strategy to combat anemia. This may take the form of formative research involving purposive data collection in selected communities where the linkages suggested here are explored in greater detail. Depending on the results, appropriate interventions (eg. livestock donations [57]) may be designed and piloted, accompanied by an impact evaluation. As Thomson, et al. [53] note, whilst much attention and donor support in Afghanistan has been paid in the last few decades to dairy and poultry sector programmes, the red meat sector has been surprisingly neglected.

## Supporting Information

S1 Supporting Information Unconditional Quantile Regression. (DOCX)

S2 Supporting Information Details of Survey Questions. (DOCX)

S1 Table Logistic regression results explaining anemia status in the AMICS sample of non-pregnant adult women in Afghanistan (n = 8270). (DOCX)

S2 Table Bonferroni robust logistic regression results explaining anemia status in the AMICS sample of adult women in Afghanistan (n = 9174). (DOCX)

S3 Table Alternative Logistic Regression Models for Anemia Status: Akaike Information Criterion and Odds Ratio for Sheep Ownership. (DOCX)
