# Supplementary material for: Reducing Anemia Prevalence in Afghanistan: Socioeconomic Correlates and the Particular Role of Agricultural Assets
Source: PLoS One. 2016 Jun 6;11(6):e0156878. doi: 10.1371/journal.pone.0156878 (PMC4894627; doi:10.1371/journal.pone.0156878)
Supplement: S1 Supporting Information — (DOCX) [file pone.0156878.s001.docx]

**S2 Supporting Information: Unconditional Quantile Regression**

Although logistic regression is commonly applied in studies exploring the drivers of nutritional status, it has important shortcomings: (i) the grouping of continuously distributed nutritional outcome variables such as Hb into small numbers of categories such as anemic and non-anemic is convenient, but is restrictive and involves a loss of statistical information, and (ii) the effects of covariates on the nutrition outcome are constrained to be the same across the distribution of the nutrition outcome.

It is logically compelling instead to model the continuous variable underlying anemia status, *i.e.* Hb, directly, but allowing for the association of covariates with Hb to vary across the distribution of Hb. Quantile regression [29] is a way to do this, and has been applied to modelling nutrition outcomes before. However, the majority of applications have estimated *conditional* quantile regression, where the association estimated between a covariate and the outcome is conditional on the values of all the other covariates, *i.e* the association is for a subgroup with particular covariate values. In terms of interpretation as well as policy-relevance, it would be desirable to have estimates that vary across the *unconditional* distribution of Hb, so that the estimated association holds for all kinds of individuals in a given part of the Hb distribution*.* We enable this by applying unconditional quantile regression based on Recentered Influence Function (RIF) regression methods [30].

**Recentered Influence Function Regression Methods**

The recentered Influence Function (RIF) regression method of estimating unconditional quantile regression (UQR) is an estimator recently introduced by Firpo and colleagues (2009) that has gained broad interest in various fields in economics. This is in large part due to the fact that the methodology shares the strengths both of OLS and quantile estimators (Jolliffe, 2011). Similarly to the quantile estimator, the UQR estimator allows the estimation of marginal effects at different points on the distribution, and in common with the OLS it respects the law of total expectations. These characteristics allow the estimation of the unconditional quantile marginal effect. In our case, this means that, for example, we can estimate the effect of sheep ownership on Hb at the 5^th^ percentile of Hb (unconditional marginal effect), rather than being restricted to estimating the effect of sheep ownership on Hb at the 5^th^ percentile of Hb, conditional on specific values of other covariates (conditional marginal effect, as estimated with the typical (conditional) quantile regression).

The RIF is built on the concept of influence functions and represents the re-centered influence of an individual observation on a distributional statistic of interest. Adding a distributional statistic, $v\left( F_{y} \right)$, to a influence function, $IF\left( Y; v, F_{y} \right)$, Firpo et al (2009) provide an estimation method based on transforming the dependent variable $(Y)$ into the RIF and then using OLS to estimate coefficients. Following Firpo et al (2009) notation, the RIF is then defined as $RIF\left( y;v \right)=v\left( F_{y} \right)+IF(y;v)$ and the conditional expectation of the RIF is modelled as a linear function of the explanatory variable

$$E\left[ RIF\left( Y;v \right)|X \right]=X\gamma+\varepsilon$$

Where the parameters $\gamma$ is estimated by an OLS. In case of quantiles, the RIF assume the following

$$RIF\left( y;Q_{t} \right)=Q_{t}+\frac{t\mathcal{-H\{}y\leq Q_{t}\}}{f_{Y}(Q_{t})}$$

Where $Q_{t}$ is the population $t-$quantile of the unconditional distribution of the dependent variable $(Y)$, $\mathcal{H}\left\{ \ldots\right\}$ an indicator function, and $f_{Y}(.)$ is the density of the marginal distribution of $Y$. The parameter estimates from the RIF regression model then provide estimates of the unconditional quantile marginal effect.

S. Firpo, N. Fortin, T. Lemieux (2009) Unconditional quantile regression Econometrica, 77 (3), pp. 953–973

D. Jolliffe, Overweight and poor? On the relationship between income and the body mass index, Economics & Human Biology, Volume 9, Issue 4, December 2011, Pages 342-355,

RIF unconditional quantile regressions were estimated for the 5^th^, 15^th^, 25^th^ and 50^th^ percentile values of the Hb distribution (the lower half of the distribution was the key focus since low Hb is of central interest). Given that the UQR models Hb directly, and thus avoids classification of individuals on the basis of anemia status, we only present a model using data unadjusted for altitudes, with the region dummy parameters partly picking up the altitude influences on Hb.

**RIF unconditional quantile regression results**

**Hemoglobin concentration: Unconditional Quantile Regression results**

|  | | | (1) | | | (2) | | | (3) | | | (4) | | |
| --- | --- | --- | --- | --- | --- | --- | --- | --- | --- | --- | --- | --- | --- | --- |
| Quantiles | | | 5% | | | 15% | | | 25% | | | 50% | | |
|  | | |  | | |  | | |  | | |  | | |
| Age | | | 0.042 | | | -0.027 | | | -0.036* | | | -0.019 | | |
|  | | | (0.043) | | | (0.032) | | | (0.021) | | | (0.019) | | |
| Age squared | | | -0.001 | | | 0.000 | | | 0.000 | | | 0.000 | | |
|  | | | (0.001) | | | (0.001) | | | (0.000) | | | (0.000) | | |
| *Education (no education as reference)* | | |  | | |  | | |  | | |  | | |
| Primary | | | -0.252 | | | -0.126 | | | 0.020 | | | 0.121 | | |
|  | | | (0.215) | | | (0.128) | | | (0.097) | | | (0.085) | | |
| Secondary plus | | | 0.082 | | | -0.203* | | | 0.007 | | | 0.160** | | |
|  | | | (0.146) | | | (0.113) | | | (0.077) | | | (0.073) | | |
| *Household head’s education (Head no education as ref.)* | | |  | | |  | | |  | | |  | | |
| Head primary | | | 0.238 | | | 0.054 | | | 0.067 | | | 0.035 | | |
|  | | | (0.174) | | | (0.116) | | | (0.077) | | | (0.067) | | |
| Head secondary plus | | | 0.116 | | | 0.015 | | | -0.012 | | | 0.016 | | |
|  | | | (0.128) | | | (0.089) | | | (0.063) | | | (0.053) | | |
| Currently pregnant | | | -0.896*** | | | -0.924*** | | | -0.680*** | | | -0.553*** | | |
|  | | | (0.251) | | | (0.152) | | | (0.111) | | | (0.076) | | |
| Recent Birth | | | -0.361** | | | -0.318*** | | | -0.173** | | | -0.185*** | | |
|  | | | (0.151) | | | (0.102) | | | (0.070) | | | (0.060) | | |
| 3+ children | | | -0.148 | | | -0.075 | | | -0.037 | | | 0.072 | | |
|  | | | (0.184) | | | (0.117) | | | (0.081) | | | (0.068) | | |
| Household size | | | -0.012 | | | -0.017 | | | -0.019** | | | -0.018*** | | |
|  | | | (0.017) | | | (0.012) | | | (0.008) | | | (0.007) | | |
| Children under 5 | | | 0.022 | | | 0.015 | | | 0.005 | | | -0.022 | | |
|  | | | (0.055) | | | (0.037) | | | (0.028) | | | (0.022) | | |
| *Language/ethnicity (Dari as ref.)* | | |  | | |  | | |  | | |  | | |
| Pashto | | | -0.025 | | | -0.174 | | | -0.207*** | | | -0.252*** | | |
|  | | | (0.147) | | | (0.108) | | | (0.071) | | | (0.062) | | |
| Uzbek | | | 0.684** | | | -0.227 | | | -0.144 | | | -0.206** | | |
|  | | | (0.302) | | | (0.210) | | | (0.125) | | | (0.093) | | |
| Turkmen | | | 1.294*** | | | 0.855*** | | | -0.050 | | | -0.483*** | | |
|  | | | (0.384) | | | (0.267) | | | (0.212) | | | (0.152) | | |
| Wealth quintile [Richest] | | |  | | |  | | |  | | |  | | |
| Poorest | | | 0.066 | | | -0.279 | | | -0.265** | | | 0.029 | | |
|  | | | (0.238) | | | (0.173) | | | (0.114) | | | (0.099) | | |
| Second | | | 0.177 | | | 0.146 | | | 0.104 | | | 0.344*** | | |
|  | | | (0.233) | | | (0.159) | | | (0.109) | | | (0.096) | | |
| Middle | | | 0.292 | | | 0.009 | | | -0.053 | | | 0.030 | | |
|  | | | (0.203) | | | (0.147) | | | (0.097) | | | (0.089) | | |
| Fourth | | | 0.191 | | | 0.188 | | | 0.105 | | | 0.155** | | |
|  | | | (0.186) | | | (0.130) | | | (0.084) | | | (0.077) | | |
|  | | | (0.0462) | | | (0.0288) | | | (0.0208) | | | (0.0177) | | |
| Drinking water is treated | | | -0.207 | | | -0.105 | | | -0.023 | | | -0.028 | | |
|  | | | (0.141) | | | (0.089) | | | (0.066) | | | (0.053) | | |
| House has electricity | | | 0.413*** | | | 0.140 | | | 0.127* | | | 0.188*** | | |
|  | | | (0.138) | | | (0.094) | | | (0.066) | | | (0.054) | | |
| Household owns agricultural land | | | 0.238* | | | 0.122 | | | 0.079 | | | 0.102* | | |
|  | | | (0.129) | | | (0.090) | | | (0.061) | | | (0.053) | | |
| Ownership of cattle | | | -0.075 | | | -0.057 | | | -0.038 | | | 0.025 | | |
|  | | | (0.155) | | | (0.107) | | | (0.075) | | | (0.063) | | |
| Ownership of Horse/donkey | | | -0.167 | | | -0.061 | | | 0.054 | | | 0.089 | | |
|  | | | (0.160) | | | (0.107) | | | (0.073) | | | (0.059) | | |
| Ownership of goat | | | 0.170 | | | 0.127 | | | 0.102 | | | 0.053 | | |
|  | | | (0.132) | | | (0.099) | | | (0.069) | | | (0.059) | | |
| Ownership of sheep | | | 0.391** | | | 0.209** | | | 0.211*** | | | 0.210*** | | |
|  | | | (0.166) | | | (0.099) | | | (0.071) | | | (0.059) | | |
| Ownership of chicken | | | -0.139 | | | -0.251*** | | | -0.113* | | | -0.044 | | |
|  | | | (0.139) | | | (0.091) | | | (0.066) | | | (0.051) | | |
| Located in rural area | | | 0.188 | | | 0.0472 | | | 0.100 | | | 0.141* | | |
|  | | | (0.175) | | | (0.120) | | | (0.0903) | | | (0.0787) | | |
| *Region (Central as reference)* | | |  | | |  | | |  | | |  | | |
| Central Highlands | | | -0.111 | | | 0.223* | | | 0.074 | | | 0.104 | | |
|  | | | (0.175) | | | (0.119) | | | (0.085) | | | (0.098) | | |
| Eastern | | | -0.297 | | | -0.841*** | | | -0.859*** | | | -1.027*** | | |
|  | | | (0.237) | | | (0.161) | | | (0.145) | | | (0.106) | | |
| Northern | | | -1.437*** | | | -1.594*** | | | -1.279*** | | | -1.809*** | | |
|  | | | (0.297) | | | (0.148) | | | (0.100) | | | (0.106) | | |
| North-eastern | | | -1.688*** | | | -1.905*** | | | -1.835*** | | | -1.744*** | | |
|  | | | (0.312) | | | (0.171) | | | (0.127) | | | (0.096) | | |
| Southern | | | -0.309* | | | -0.125 | | | -0.106 | | | -0.344*** | | |
|  | | | (0.184) | | | (0.144) | | | (0.097) | | | (0.094) | | |
| South-eastern | | | -0.863*** | | | -0.851*** | | | -0.715*** | | | -0.733*** | | |
|  | | | (0.211) | | | (0.135) | | | (0.107) | | | (0.094) | | |
| Western | | | -1.574*** | | | -0.950*** | | | -0.610*** | | | -0.827*** | | |
|  | | | (0.324) | | | (0.145) | | | (0.096) | | | (0.094) | | |
| Constant | | | 9.858*** | | | 13.086*** | | | 13.640*** | | | 14.337*** | | |
|  | | | (0.647) | | | (0.489) | | | (0.316) | | | (0.289) | | |
|  | | |  | | |  | | |  | | |  | | |
| R-squared | | | 0.025 | | | 0.063 | | | 0.101 | | | 0.136 | | |
|  | | |  | | |  | | |  | | |  | | |

Regression estimates with standard errors in parentheses. Covariates also include a dummy variable to capture a small number of observations with missing information for language/ethnicity. Covariates also include a dummy variable to capture a small number of observations that either had missing information for language/ethnicity or spoke a language other than Dari, Pashto, Uzbek or Turkmen. *** denotes statistical significance at the 1% level, ** at 5% level and * at 10% level.
