# Supplementary material for: Reducing Anemia Prevalence in Afghanistan: Socioeconomic Correlates and the Particular Role of Agricultural Assets
Source: PLoS One. 2016 Jun 6;11(6):e0156878. doi: 10.1371/journal.pone.0156878 (PMC4894627; doi:10.1371/journal.pone.0156878)
Supplement: S2 Supporting Information — (DOCX) [file pone.0156878.s002.docx]

**S5 Supporting Information: Details of Survey Questions**

**AMICS 2010-11**

All questionnaires are publicly available at the end of the AMIC’s final report, which can be downloaded from Afghanistan’s Central Statistics Organization’s webpage at <http://cso.gov.af/Content/files/AMICS.pdf>. The variables included in the models are based on the following questions and sections of the survey.

| **Variable** | **Questionnaire** | **Section** | **Question(s)** |
| --- | --- | --- | --- |
| Haemoglobin level & anaemia | Individual women | Women aged 15-49 selected for blood test (SCW) | SCW7 |
| Age | Individual women | Woman's background (WB) | WB2 |
| Woman's education |  |  | WB4 |
| Pregnancy status | Individual women | Contraception (CP) | CP1 |
| High parity | Individual women | Child mortality (CM) | CM11 |
| Recent birth |  |  | CM13 |
| Place of residence is rural | Household | Household information panel (HH) | HH6 |
| Region of residence |  |  | HH7 |
| Household size |  |  | HH11 |
| No. of children under 5 |  |  | HH14 |
| Head of household's education | Household | Education (ED) | ED4* |
| Ethnicity |  | Household characteristics (HC) | HC1B |
| Electricity in the household | Household | Household characteristics (HC) | HC8 [A] |
| Household owns agricultural land |  |  | HC11 |
| Household owns farm animals |  |  | HC14 [A-E] |
| Drinking water is treated | Household | Water & sanitation (WS) | WS6 |
| Wealth quintile | Household |  | windex5* |
| **The variables measuring the household head's educational attainment and household's wealth score are readily available in the household's database and are named helevel and windex5, respectively. They however do not appear as such in the questionnaire. The former one must have been created based on question ED4 in the household questionnaire ("What is the highest level of school the household head attended"). The variable ED4 is included in the Household Members database but not in the Household one, which is the one used for this analysis. The wealth score in turn was created by the MICS team based on several questions from the household's questionnaire. These are: the main source of water in the household, and the type of sanitation facility and cooking fuel; the main material of roofs, floors, and walls; the ratio of people to sleeping rooms; and ownership of refrigerator, TV and radio.* | | | |
|  |  |  |  |
|  |  |  |  |
|  |  |  |  |

NRVA 2011-12

The full NRVA questionnaires are publicly available from the International Household Survey Network (IHSN) (<http://catalog.ihsn.org/index.php/catalog/5230>). A shorter form of the questionnaires can also be found in the Appendix of the NRVA 2011-12 Report^^[[1]](#footnote-1)^^.

The variables included in the models are based on the following questions and sections of the survey

| Variable | Questionnaire | Section | Question(s) |
| --- | --- | --- | --- |
| Age household head | Household | 3 | 3.5 |
| Level of education household head | Household | 12 | 12.3 |
| Dependency ratio | Household | 3 | 3.5 |
| Males | Household | 3 | 3.4 – 3.5 |
| Females | Household | 3 | 3.4 – 3.5 |
| Kids | Household | 3 | 3.4 – 3.5 |
| Rural | Household | 1 | 1.2 |
| Ramadam | Household | 2 | 2.1 |
| Consumption (Calories) | Household (female) | 23 | 23.1-23.6 |
| Wealth | Household | 4 | 4.1-4.6 |
| Market in the community | Household (Shura) | 3 | 3.18 |
| Consumption of mutton (dummy) | Household (female) | 23 | 23_4 |
| Consumption of mutton (grams) | Household (female) | 23 | 23_6 |
| Consumption of mutton (days) | Household (female) | 23 | 23_4 |
| Source consumption of mutton | Household | 23 | 23_5 |
| Ownership of sheep | Household | 5 | 5.2 |
| Time of interview | Household | 2 | 2.1 |
| Province dummies | Household | 1 | 1.3 |

1. Central Statistics Organisation (2014). "National Risk and Vulnerability Assessment (NRVA) 2011-12. Afghanistan Living Condition Survey." CSO, Kabul (Afghanistan). Online at http://www.af.undp.org/content/dam/afghanistan/docs/MDGs/NRVA%20REPORT-rev-5%202013.pdf [↑](#footnote-ref-1)
