# Supplementary material for: Reducing Anemia Prevalence in Afghanistan: Socioeconomic Correlates and the Particular Role of Agricultural Assets
Source: PLoS One. 2016 Jun 6;11(6):e0156878. doi: 10.1371/journal.pone.0156878 (PMC4894627; doi:10.1371/journal.pone.0156878)
Supplement: S1 Table — (DOCX) [file pone.0156878.s003.docx]

**S1 Table: Logistic regression results explaining anemia status in the AMICS sample of non-pregnant adult women in Afghanistan (n=8270)**

|  | Anemic (unadjusted) | | Anemic (adjusted) | |
| --- | --- | --- | --- | --- |
|  | Odds Ratio | Std. Err. | Odds Ratio | Std. Err. |
| Age in years | 1.041 | 0.025 | 1.041* | 0.022 |
| Age in years squared | 0.999 | 0.000 | 0.999* | 0.000 |
| *Education (No schooling as reference)* |  |  |  |  |
| Primary schooling | 1.066 | 0.109 | 1.037 | 0.096 |
| Secondary + schooling | 0.980 | 0.104 | 0.988 | 0.088 |
| *Household head's education (Head no education as reference)* |  |  |  |  |
| Head primary | 0.946 | 0.091 | 1.015 | 0.079 |
| Head secondary plus | 0.999 | 0.077 | 1.118* | 0.066 |
| Gave birth in last two years | 1.184** | 0.078 | 1.191** | 0.069 |
| Has 3+ children | 1.188* | 0.096 | 1.068 | 0.083 |
| Number of household members | 1.023** | 0.009 | 1.030*** | 0.008 |
| Number of under-5s in household | 0.948* | 0.031 | 0.962 | 0.027 |
| *Language/ethnicity (Dari as reference)* |  |  |  |  |
| Pashto speaker | 1.304*** | 0.088 | 1.161** | 0.075 |
| Uzbek speaker | 1.183 | 0.103 | 1.281** | 0.099 |
| Turkmen speaker | 1.017 | 0.171 | 1.900*** | 0.162 |
| *Wealth quintiles (quintile 1 as reference)* |  |  |  |  |
| Wealth quintile 2 | 0.709*** | 0.091 | 0.586*** | 0.081 |
| Wealth quintile 3 | 0.795** | 0.099 | 0.629*** | 0.086 |
| Wealth quintile 4 | 0.777** | 0.111 | 0.580*** | 0.098 |
| Wealth quintile 5 | 0.707** | 0.144 | 0.547*** | 0.125 |
| Drinking water is treated | 1.103 | 0.076 | 1.093 | 0.065 |
| House has electricity | 0.887 | 0.077 | 0.822*** | 0.066 |
| Household owns agricultural land | 0.938 | 0.070 | 0.922 | 0.061 |
| Household owns cattle | 1.018 | 0.082 | 1.047 | 0.071 |
| Household owns horses/donkeys | 1.004 | 0.079 | 0.971 | 0.069 |
| Household owns goats | 0.896 | 0.077 | 0.826*** | 0.068 |
| Household owns sheep | 0.825** | 0.079 | 0.855** | 0.069 |
| Household owns chicken | 1.182** | 0.071 | 1.047 | 0.062 |
| Located in rural area | 0.921 | 0.104 | 0.939 | 0.090 |
| *Region (Central as reference)* |  |  |  |  |
| Located in Central Highlands | 0.594** | 0.203 | 1.346** | 0.125 |
| Located in Eastern Region | 2.216*** | 0.144 | 0.876 | 0.127 |
| Located in Northwest Region | 4.090*** | 0.114 | 2.332*** | 0.095 |
| Located in Northeastern Region | 5.853*** | 0.117 | 2.727*** | 0.099 |
| Located in Southern Region | 1.420** | 0.144 | 1.255** | 0.109 |
| Located in Southeastern Region | 2.320*** | 0.127 | 1.984*** | 0.103 |
| Located in Western Region | 2.549*** | 0.131 | 1.241* | 0.112 |
| Intercept | 0.596*** | 0023 | 0.188*** | 0.062 |
| *Observations* | 8270 |  | 8270 |  |
| *Hosmer-Lemeshow Chi-squared test statistic (675)* | 629.82 |  | 715.16 |  |
| *Hosmer-Lemeshow p-value* | *0.882* |  | *0.126* |  |

Logistic regression odds ratios for anemia status based on altitude-adjusted and unadjusted haemoglobin values. Covariates also include a dummy variable to capture a small number of observations with missing information for language/ethnicity. Std. Err. denotes robust standard errors. *** denotes statistical significance at the 1% level, ** at 5% level and * at 10% level.
