# Supplementary material for: Reducing Anemia Prevalence in Afghanistan: Socioeconomic Correlates and the Particular Role of Agricultural Assets
Source: PLoS One. 2016 Jun 6;11(6):e0156878. doi: 10.1371/journal.pone.0156878 (PMC4894627; doi:10.1371/journal.pone.0156878)
Supplement: S2 Table — (DOCX) [file pone.0156878.s004.docx]

**S3 Table. Bonferroni robust logistic regression results explaining anemia status in the AMICS sample of adult women in Afghanistan (n=9174)**

|  | Anemic (unadjusted) | | Anemic (adjusted) | |
| --- | --- | --- | --- | --- |
|  | Odds ratio | p-value | Odds ratio | p-value |
| Age in years | 1.040 | 1.000 | 1.043 | 1.000 |
| Age in years squared | 1.000 | 1.000 | 0.999 | 1.000 |
| *Education (No schooling as reference)* |  |  |  |  |
| Primary schooling | 1.114 | 1.000 | 1.089 | 1.000 |
| Secondary + schooling | 1.007 | 1.000 | 1.040 | 1.000 |
| *Household head's education (Head no education as reference)* |  |  |  |  |
| Head primary | 0.949 | 1.000 | 1.006 | 1.000 |
| Head secondary plus | 1.001 | 1.000 | 1.114 | 1.000 |
| Currently Pregnant | 0.846 | 1.000 | 0.884 | 1.000 |
| Gave birth in last two years | 1.256 | 0.069 | 1.220 | 0.069 |
| Has 3+ children | 1.071 | 1.000 | 1.008 | 1.000 |
| Number of household members | 1.017 | 1.000 | 1.028 | 0.009 |
| Number of under-5s in household | 0.955 | 1.000 | 0.984 | 1.000 |
| *Language/ethnicity (Dari as reference)* |  |  |  |  |
| Pashto speaker | 1.357 | 0.008 | 1.178 | 0.776 |
| Uzbek speaker | 1.127 | 1.000 | 1.211 | 1.000 |
| Turkmen speaker | 0.979 | 1.000 | 1.850 | 0.004 |
| *Wealth quintiles (quintile 1 as reference)* |  |  |  |  |
| Wealth quintile 2 | 0.750 | 0.028 | 0.594 | 0.000 |
| Wealth quintile 3 | 0.795 | 0.477 | 0.615 | 0.000 |
| Wealth quintile 4 | 0.775 | 0.559 | 0.563 | 0.000 |
| Wealth quintile 5 | 0.702 | 0.354 | 0.520 | 0.000 |
| Drinking water is treated | 1.123 | 1.000 | 1.113 | 1.000 |
| House has electricity | 0.944 | 1.000 | 0.855 | 0.444 |
| Household owns agricultural land | 0.903 | 1.000 | 0.897 | 1.000 |
| Household owns cattle | 0.998 | 1.000 | 1.028 | 1.000 |
| Household owns horses/donkeys | 1.018 | 1.000 | 0.950 | 1.000 |
| Household owns goats | 0.922 | 1.000 | 0.839 | 0.214 |
| Household owns sheep | 0.802 | 0.104 | 0.830 | 0.140 |
| Household owns chicken | 1.193 | 0.326 | 1.070 | 1.000 |
| Located in rural area | 0.969 | 1.000 | 0.969 | 1.000 |
| *Region (Central as reference)* |  | 0.355 |  | 0.192 |
| Located in Central Highlands | 0.603 | 0.000 | 1.398 | 1.000 |
| Located in Eastern Region | 2.278 | 0.000 | 0.887 | 0.000 |
| Located in Northwest Region | 4.163 | 0.000 | 2.358 | 0.000 |
| Located in Northeastern Region | 5.939 | 0.539 | 2.735 | 0.486 |
| Located in Southern Region | 1.396 | 0.000 | 1.292 | 0.000 |
| Located in Southeastern Region | 2.678 | 0.000 | 2.253 | 1.000 |
| Located in Western Region | 2.503 | 0.355 | 1.207 | 0.192 |

Estimation details are identical to those listed below Table 4.
