# Supplementary material for: Reducing Anemia Prevalence in Afghanistan: Socioeconomic Correlates and the Particular Role of Agricultural Assets
Source: PLoS One. 2016 Jun 6;11(6):e0156878. doi: 10.1371/journal.pone.0156878 (PMC4894627; doi:10.1371/journal.pone.0156878)
Supplement: S3 Table — (DOCX) [file pone.0156878.s005.docx]

**S4 Table: Alternative Logistic Regression Models for Anemia Status: Akaike Information Criterion and Odds Ratio for Sheep Ownership**

| Anemic (unadjusted) | I | II | III | IV | V | VI | VII | VIII | IX |
| --- | --- | --- | --- | --- | --- | --- | --- | --- | --- |
| Household owns sheep | 0.859*** | 0.843*** | 0.832*** | 0.761*** | 0.775*** | 0.766*** | 0.765*** | 0.760*** | 0.802*** |
|  | (0.057) | (0.058) | (0.058) | (0.062) | (0.062) | (0.063) | (0.063) | (0.071) | (0.074) |
| Mother's characteristics |  | Y | Y | Y | Y | Y | Y | Y | Y |
| Head of household characteristics |  |  | Y | Y | Y | Y | Y | Y | Y |
| Household characteristics |  |  |  | Y | Y | Y | Y | Y | Y |
| Language/ethnicity |  |  |  |  | Y | Y | Y | Y | Y |
| Wealth quintiles |  |  |  |  |  | Y | Y | Y | Y |
| Infrastructure |  |  |  |  |  |  | Y | Y | Y |
| Other agricultural assets |  |  |  |  |  |  |  | Y | Y |
| Regional dummies |  |  |  |  |  |  |  |  | Y |
| AIC | 9275 | 9229 | 9224 | 9207 | 9070 | 9060 | 9061 | 9064 | 8639 |

| Anemic (adjusted) | I | II | III | IV | V | VI | VII | VIII | IX |
| --- | --- | --- | --- | --- | --- | --- | --- | --- | --- |
| Household owns sheep | 0.938 | 0.923 | 0.917* | 0.812*** | 0.824*** | 0.814*** | 0.808*** | 0.882** | 0.830*** |
|  | (0.050) | (0.051) | (0.051) | (0.055) | (0.055) | (0.055) | (0.055) | (0.063) | (0.065) |
| Mother's characteristics |  | Y | Y | Y | Y | Y | Y | Y | Y |
| Head of household characteristics |  |  | Y | Y | Y | Y | Y | Y | Y |
| Household characteristics |  |  |  | Y | Y | Y | Y | Y | Y |
| Language/ethnicity |  |  |  |  | Y | Y | Y | Y | Y |
| Wealth quintiles |  |  |  |  |  | Y | Y | Y | Y |
| Infrastructure |  |  |  |  |  |  | Y | Y | Y |
| Other agricultural assets |  |  |  |  |  |  |  | Y | Y |
| Regional dummies |  |  |  |  |  |  |  |  | Y |
| AIC | 11119 | 11082 | 11083 | 11037 | 10945 | 10895 | 10888 | 10883 | 10676 |

Note: Mother's characteristics includes age, age squared, education dummies, currently pregnant, gave birth in last two years, has 3+ children; head of household characteristics includes education dummies; household characteristics includes number of household members, number of under-5s in household, rural; language/ethnicity includes language dummies, wealth quintiles includes wealth dummies; infrastructure includes drinking water is treated, house has electricity; other agricultural assets includes household owns agricultural land, household owns cattle, household owns horses/donkeys, household owns goats, household owns chicken, regional dummies includes regional dummies.
